# Dynamic contrast-enhanced magnetic resonance imaging for monitoring neovascularization during bone regeneration—a randomized in vivo study in rabbits

**DOI:** 10.1007/s00784-021-03889-6

**Published:** 2021-03-30

**Authors:** L. A. R. Righesso, M. Terekhov, H. Götz, M. Ackermann, T. Emrich, L. M. Schreiber, W. E. G. Müller, J. Jung, J. P. Rojas, B. Al-Nawas

**Affiliations:** 1 Clinic for Oral and Maxillofacial Surgery and Plastic Surgery, University Medical Center of the Johannes Gutenberg University Mainz, Augustusplatz 2, 55131 Mainz, Germany; 2 Molecular and Cellular Imaging, Comprehensive Heart Failure Center, University Hospital Würzburg, Josef-Schneider-Strasse 2, 97080 Würzburg, Germany; 3 Cell Biology Unit, University Medical Center of the Johannes Gutenberg University Mainz, Langenbeckstrasse 1, 55131 Mainz, Germany; 4 Institute of Functional and Clinical Anatomy, University Medical Center of the Johannes Gutenberg University Mainz, Johann-Joachim-Becher-Weg 13, 55128 Mainz, Germany; 5 Department of Radiology, University Medical Center of the Johannes Gutenberg University Mainz, Langenbeckstrasse 1, 55131 Mainz, Germany; 6 Medical University of South Carolina, 171 Ashley Avenue, Charleston, SC 29425 USA; 7 German Center for Cardiovascular Research (DZHK), Partner-Site Rhine-Main, Potsdamer Strasse 58, 10785 Berlin, Germany; 8 Institute of Physiological Chemistry, University Medical Center of the Johannes Gutenberg University Mainz, Duesbergweg 6, 55128 Mainz, Germany; 9 Department of Oral and Maxillofacial Surgery, School of Dentistry, Kyung Hee University, 23, Kyung Hee Dae-ro, Dongdaemun-gu, Seoul, 02447 Republic of Korea; 10 Private Practice, Av. La Dehesa, 181 Santiago, Chile

**Keywords:** Animal experimentation, Bone regeneration, Multiparametric magnetic resonance imaging, Neovascularization, physiologic, Tissue engineering, Translational medical research

## Abstract

**Objectives:**

Micro-computed tomography (μ-CT) and histology, the current gold standard methods for assessing the formation of new bone and blood vessels, are invasive and/or destructive. With that in mind, a more conservative tool, dynamic contrast-enhanced magnetic resonance imaging (DCE-MRI), was tested for its accuracy and reproducibility in monitoring neovascularization during bone regeneration. Additionally, the suitability of blood perfusion as a surrogate of the efficacy of osteoplastic materials was evaluated.

**Materials and methods:**

Sixteen rabbits were used and equally divided into four groups, according to the time of euthanasia (2, 3, 4, and 6 weeks after surgery). The animals were submitted to two 8-mm craniotomies that were filled with blood or autogenous bone. Neovascularization was assessed in vivo through DCE-MRI, and bone regeneration, ex vivo, through μ-CT and histology.

**Results:**

The defects could be consistently identified, and their blood perfusion measured through DCE-MRI, there being statistically significant differences within the blood clot group between 3 and 6 weeks (*p* = 0.029), and between the former and autogenous bone at six weeks (*p* = 0.017). Nonetheless, no significant correlations between DCE-MRI findings on neovascularization and μ-CT (*r* =−0.101, 95% CI [−0.445; 0.268]) or histology (*r* = 0.305, 95% CI [−0.133; 0.644]) findings on bone regeneration were observed.

**Conclusions:**

These results support the hypothesis that DCE-MRI can be used to monitor neovascularization but contradict the premise that it could predict bone regeneration as well.

## Introduction

Tissue engineering is based on three pillars: reparative cells that can form a functional matrix; an appropriate scaffold for transplantation and support; and bioreactive molecules, like cytokines and growth factors, which can support and coordinate the formation of the desired tissue [1]. However, more recent studies show that, without the establishment of a vascular network, incorporation of engineered constructs simply does not take place [2]. Hence, research on new ways of improving construct vascularity is gaining momentum [3–5].

Many of the studies on the topic, however, rely on invasive and/or destructive tools, such as histology and radiation-emitting imaging, to assess bone regeneration and neovascularization [6, 7]. Though accurate, these methods present a few downsides. Histology, for one, requires a surgical specimen. In clinical studies, when it is possible to obtain one, additional harm is usually caused to patients, rendering repeated measurements virtually impossible. As for animal studies, subjects must be euthanized, precluding an intra-individual longitudinal assessment. Alternatively, an inter-individual analysis could be performed, but that would imply sacrificing animals at each time point, increasing sample size and, consequently, costs, but more importantly, clashing with the 3Rs of animal research (replacement, reduction, and refinement). Moreover, for histological analysis, the surgical specimen must be sliced, rendering further examinations by other methods difficult or impossible. Lastly, results are reported by sections and, for volumetric determinations, the results are often estimated by interpolation [8].

More recently, micro-computed tomography (μ-CT) has been described for monitoring neovascularization and bone regeneration [9]. Among its advantages, it can be cited that it not only allows assessing both outcomes but also allows doing so three-dimensionally. Moreover, it is a non-destructive method [10]. Nevertheless, the ionizing radiation emitted during μ-CT imaging becomes hazardous when research subjects need to be submitted to repeated exams [11]. Also, in many instances, similarly to histologic analysis, to perform a μ-CT, a surgical specimen must be obtained and the animal euthanized, which also precludes an intra-individual longitudinal assessment.

As a non-invasive imaging technique, magnetic resonance imaging (MRI) is suitable for in vivo longitudinal evaluation of tissue repair following grafting procedures. Non-invasive dynamic MRI following the administration of a contrast agent can provide functional information on the microcirculation. This technique, also known as dynamic contrast-enhanced MRI (DCE-MRI), has shown potential in a wide range of clinical applications, especially in oncology, where physiological properties, such as blood flow, blood volume, and vessel permeability need to be determined quantitatively [12]. However, despite its well-established role in assessing the microvasculature in pre- and clinical and studies, the use of DCE-MRI in tissue engineering has been little explored.

The aim of the present study was to assess the accuracy and reproducibility of DCE-MRI in monitoring neovascularization during bone regeneration and to evaluate whether the initial area under the contrast concentration versus time curve at 140 s (IAUC_140_), a DCE-MRI pharmacokinetic parameter of blood perfusion, is a reliable surrogate of the efficacy of osteoplastic materials.

## Materials and methods

This research was developed in an exploratory experimental design, with randomized sampling, a control group, and blinded evaluators.

The experiment complied with the fundamental principles of good laboratory practice, having received ethical approval from the Tierschutzkommission Rheinland-Pfalz (G 15-1-084).

### Population and sample

For this study, 16 skeletally mature (5–6 months old) New Zealand white female rabbits, weighing 3.5–4.5 kg, were used. Animals were randomly divided into four groups of four, according to the time of euthanasia (2, 3, 4, and 6 weeks after surgery), using an online free calculator (www.graphpad.com/quickcalcs/randomize1/, GraphPad Software, San Diego, USA).

### Facilities

Throughout the experiment, animals were housed under satisfactory conditions, in closed, ventilated shelves, with filtered airflow, temperature, and humidity control, under cold light and supervision of veterinary staff. They were fed solid ration, appropriate to them, and water ad libitum. They were kept in standardized cages, labeled, and identified with the group number to which they belonged, date of birth, weight, date of surgery, date of death, names of researcher, and advisor. Cages were cleaned three times a week, with running water, soap, and disinfectant.

### Surgical procedure

Surgery was performed under rigorous aseptic conditions and general anesthesia. The latter was obtained with an intramuscular (IM) injection of a combination of medetomidine (0.2 mg/kg), midazolam (1 mg/kg), and fentanyl (0.02 mg/kg).

Once the animal was unconscious, the surgical site was shaved, washed with 2% iodine antiseptic solution and then, infiltrated with 1.8 ml of 2% lidocaine HCL with 1:100.000 epinephrine. A coronal incision was made, and the soft tissues reflected to expose the calvarium. Using a surgical handpiece with a diamond trephine, while irrigating with copious physiological saline, two craniotomies (8 mm diameter) were prepared in the parietal bones. Care was taken not to tear the dura mater.

**Fig. 1:**
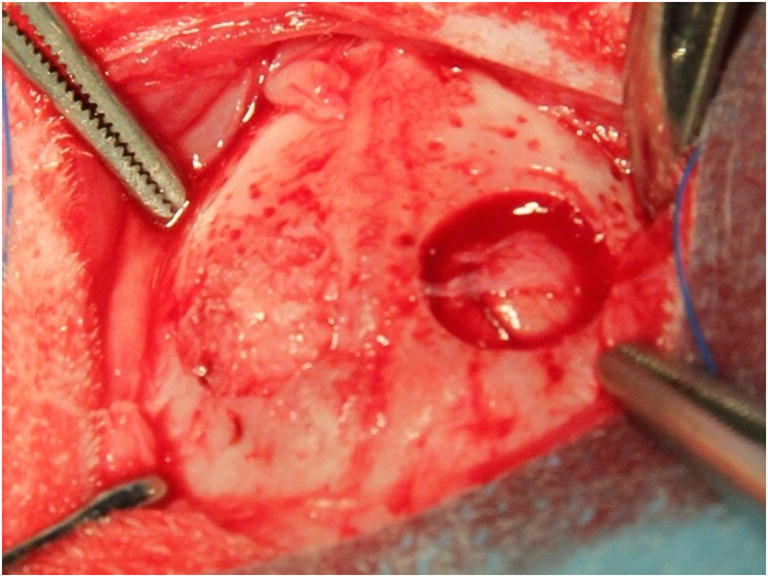
Craniotomies prepared in the parietal bones. On the left side, the defect was grafted with autogenous bone, while on the right side, it was left to fill with blood

One defect was grafted, until visually filled, with autogenous bone chips, obtained through the morselization of the trephined bone with a bone scraper (Geistlich SafeScraper TWIST, Geistlich Pharma AG, Wolhusen, Switzerland), while the other defect was left to fill with blood and served as control (Fig. 1).

Treatments were allocated to the craniotomies, according to a 4 × 2 Latin squares design. Next, a resorbable, collagen membrane (Geistlich Bio-Gide®, Geistlich Pharma AG, Wolhusen, Switzerland) was used to protect the initial coagulum and prevent particle migration or soft tissue ingrowth into the grafted sites. Finally, soft tissues were approximated and sutured in layers.

Immediately after surgery, the animals were administered subcutaneous injections of the antibiotic enrofloxacin 2.5% (0.4 ml/kg), the anti-inflammatory carprofen 50 mg/ml (0.1 ml/kg), and the opioid analgesic buprenorphine (0.07 ml/kg). This was repeated once a day, for the next 3 days.

### Dynamic contrast-enhanced magnetic resonance imaging

**Fig. 2:**
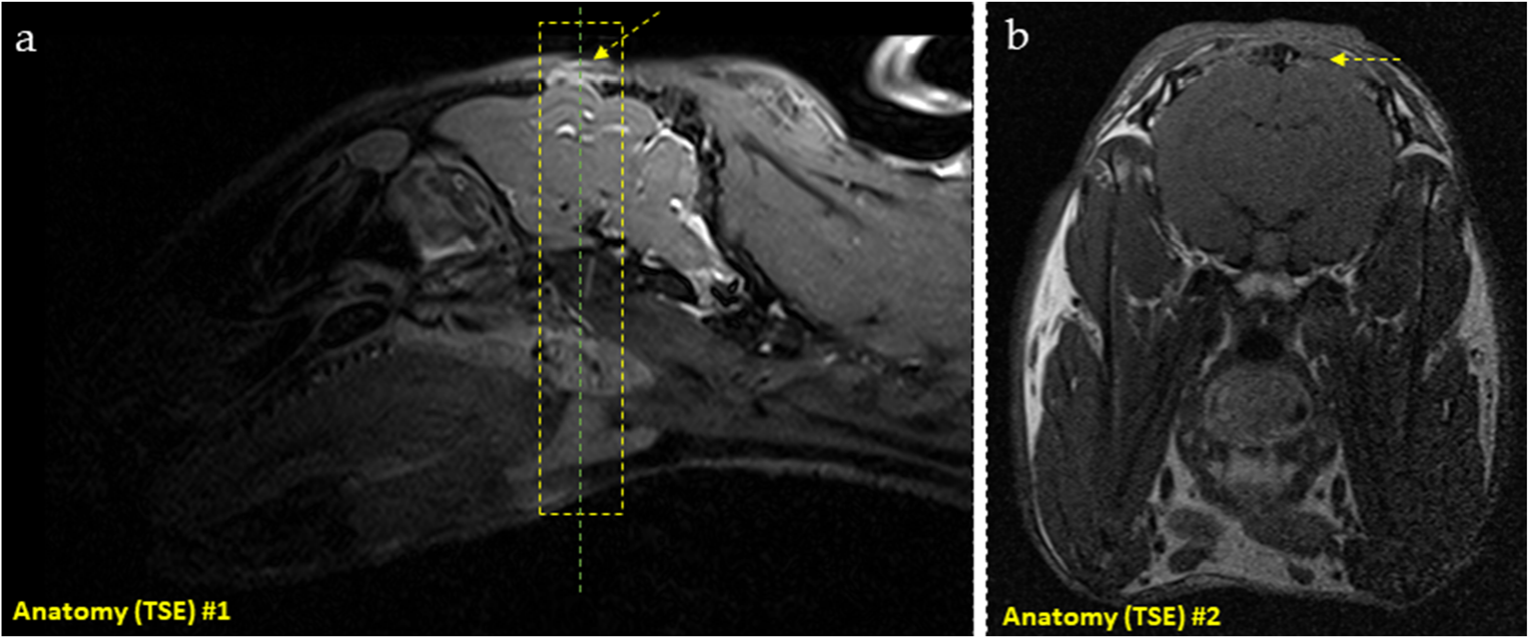
**a** Sagittal slice of high-resolution anatomical image delineating one of the surgical sites. The yellow rectangle represents the transversal slab used for planning the DCE-MRI slices. **b** Coronal slice of a high-resolution anatomical image used to confirm localization. This slice corresponds to the dashed line at the center of the rectangle in a. The yellow arrow identifies a specific point that is shown in two different planes

Image acquisition was performed under general anesthesia, using the same protocol as for surgery. Imaging sessions were held at 2, 3, 4, and 6 weeks after surgery, one group at a time, according to the established time of euthanasia. The device used was the 3-Tesla MAGNETOM® Prisma scanner (Siemens Healthineers GmbH, Erlangen, Germany), with a 4-channel flexible receive array and a body transmit coil. Animals were placed in prone position and an axial orientation was used for all the slices. Overview proton-density contrast images in the sagittal plane were acquired to plan slices for the subsequent DCE-MRI. The pulse sequence used was turbo spin-echo, with TR/TE = 1100/39 ms, echo train length = 29, slice thickness = 0.5 mm, number of slices = 96, image matrix = 448 × 275, and field-of-view = 219 × 150 mm. Additionally, high-resolution anatomical images in the transversal plane were acquired at the operated area using a turbo spin-echo sequence, with TR/TE = 600/15 ms, field-of-view = 89 × 89 mm, matrix 438 × 336, voxel size=0.2 × 0.25 × 1.5 mm, and 16 slices covering the operated area (Fig. 2). (For more details, please refer to Table 1).

**Table 1:** Parameters of measurement protocols

Parameter	Anatomy (TSE)	Anatomy (TSE)	Perfusion (GRE 3D)
Orientation	Transversal	Sagittal	Transversal
Repetition time (ms)	600	1100	15
Echo train length	4	29	N/A
Echo time (ms)	15	39	1.79
Matrix size	356 × 448	356 × 448	128 × 128
Slice thickness (mm)	1.5	0.5	1
Field-of-view (mm)	89 × 89	150 × 219	62 × 62
Number of slices	16	96	10
Measurements	1	1	20

Anatomy protocols (turbo spin-echo columns 1 and 2) used for localization of the slab. Column #3 provides parameters for perfusion data acquisition

Dynamic T1-weighted images were acquired using a fast SGRE pulse sequence, with TR/TE = 15/1.8 ms, Flip Angle = 750, image matrix = 128 × 128, field-of-view = 62 × 62 mm, voxel size = 0.48 × 0.48 × 1 mm, number of slices =10, and a parallel imaging acceleration factor *R*=2. After the acquisition of five baseline images, gadolinium diethylenetriamine pentaacetic acid (Gd-DTPA) was administered as a rapid bolus (0.1 mmol of Gd/kg) through the ear vein and imaging continued for 6 min post-injection with an effective temporal resolution of 8.2 s.

After the imaging session, still under general anesthesia, the animals were sacrificed through an anesthetic overdose. The calvarium was excised and placed in 10% formalin solution for subsequent μ-CT and histological procedures.

Perfusion analysis was performed by an experienced radiologist (T.E.), using a dedicated software solution CVI42® (Circle Cardiovascular Imaging Inc., Calgary, Canada). Initially, high-resolution T2 images were used to locate the defects and their borders. Then, these anatomical images were matched to the perfusion datasets in the same software to allow optimal contouring of the region of interest (ROI). Contours were drawn manually for both defects, as well as for the reference tissues, the brain at brain stem level, and the right masseter muscle. Manual contouring was performed for every slice and time point in the perfusion dataset. Every defect consisted of ten slices, which included both defects in coronal angulation. In questionable cases, a consensus ROI was drawn after interdisciplinary discussion (T.E. and L.A.R.R.). Motion and artifacts were carefully excluded. Furthermore, potential confounders of perfusion, like vessels, were carefully eliminated. Perfusion curves and measurement included the relative signal intensity over time.

The initial area under the curve at 140 s (IAUC_140_) was then calculated for each ROI (left defect, right defect, brain tissue, muscle tissue) on each slice using IBM SPSS Statistics for Windows, version 23.0 (IBM Corp., Armonk, NY, USA). Finally, a mean value was established for each ROI.

### Micro-computed tomography

Imaging was performed using the high-resolution micro-computed tomography scanner μ-CT 40 (Scanco Medical AG, Brüttisellen, Switzerland). Samples containing the whole calvarium were scanned under the following specifications: the specimen tube of the μ-CT scanner was run with the X-ray tube operating at 70 kVp and 114 μA, with a focal spot size of 6 μm. Per rotation, 1000 pictures were taken with an integration time of 300 ms and 2048 × 2048 pixels. Reconstruction resulted in 208 pictures/probe with a voxel size of 18 μm, corresponding to a layer thickness of 3 μm and height of 6 mm. Thereby, the whole calvaria could be reconstructed. Bone volume fraction (BVF) was then computed by dividing the bone volume by a specified cylindrical total volume, defined by the initial drilling diameter (8 mm) and the thickness of the calvaria. This cylinder was orthogonally projected onto the bone defect and the calculation was performed using the Scanco Medical Software (Scanco Medical AG, Brüttisellen, Switzerland).

### Histological analysis

After μ-CT, the samples were prepared for histological analysis. Mineralized samples were directly embedded in Technovit® 7200 VLC (Kulzer GmbH, Hanau, Germany). Sectioning and grinding of Technovit® embedded samples were performed as described by Donath [13]. The slices were then stained with toluidine blue and analyzed under conventional qualitative bright-field microscopy analysis. For quantification of new bone formation (NBF), the quantity of newly formed bone was calculated as the percentage fraction of the new bone area to the total defect area. A histomorphometric analysis of the tissue area on the pictures was performed using ImageJ (NIH). Prior to that, the pictures were anonymized using the extension AbleBits.com random generator, for Microsoft® Excel® 2013 (Microsoft Corporation, Redmond, USA), so the evaluator could assess them blindly.

### Statistical analysis

Data were analyzed using IBM SPSS Statistics for Windows, version 23.0 (IBM Corp., Armonk, NY, USA). The bone defect was set as the statistical unit. A generalized estimating equation test was used to compare treatment and time of euthanasia using exchangeable correlation structure, accounting for correlations between repeated measurements on the same animal. Interactions were included in the model, if necessary, and mean values were compared using simple effects analyses with a post hoc Bonferroni adjustment. Correlations between μ-CT and DCE-MRI, histology, and DCE-MRI, μ-CT, and histology were assessed using Spearman’s rank correlation coefficient.

**Fig. 3:**
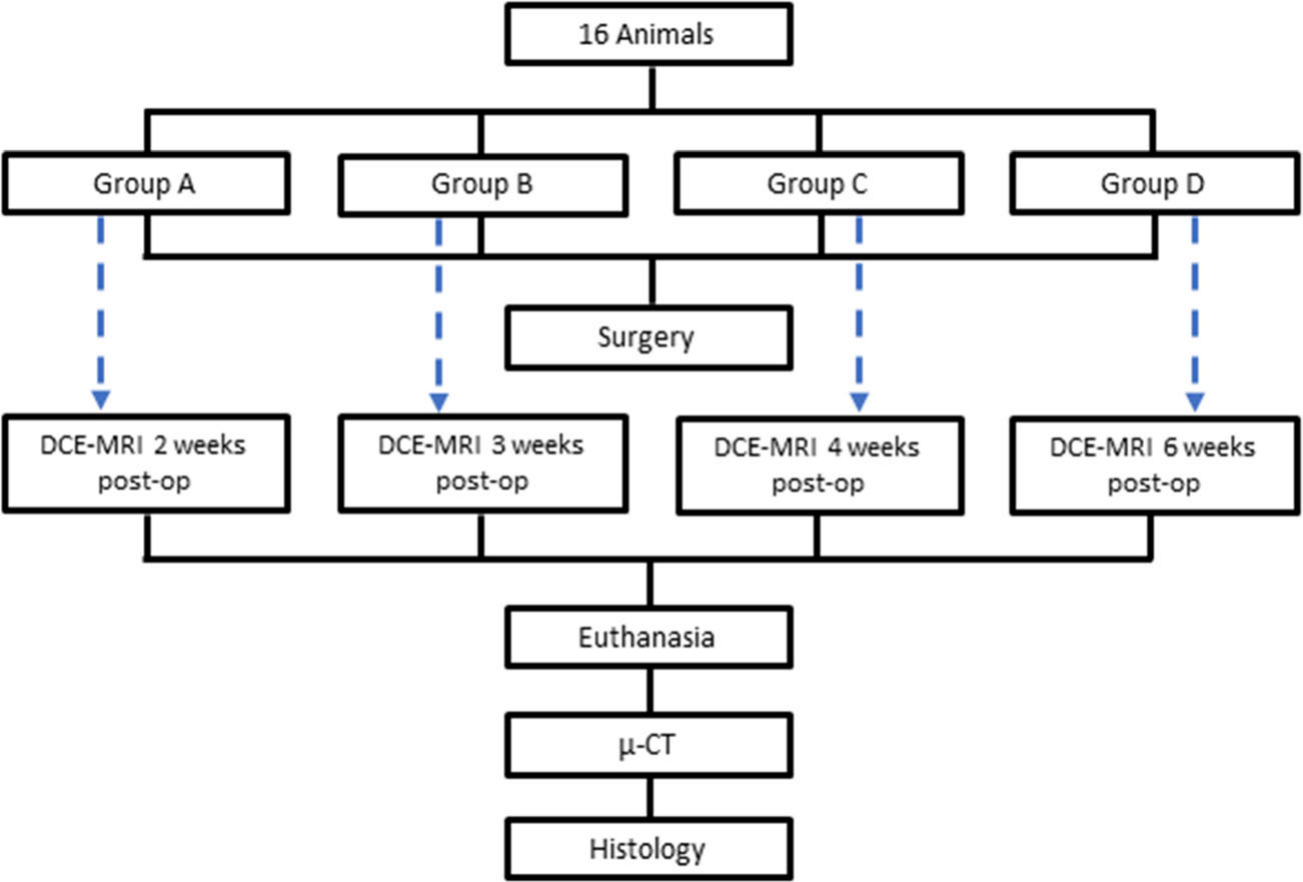
Flow chart of the study design

In Fig. 3, the study design is summarized in graphical form to facilitate comprehension, as recommended in the ARRIVE guidelines [14].

## Results

### Dynamic contrast-enhanced magnetic resonance imaging

#### Descriptive analysis

**Fig. 4:**
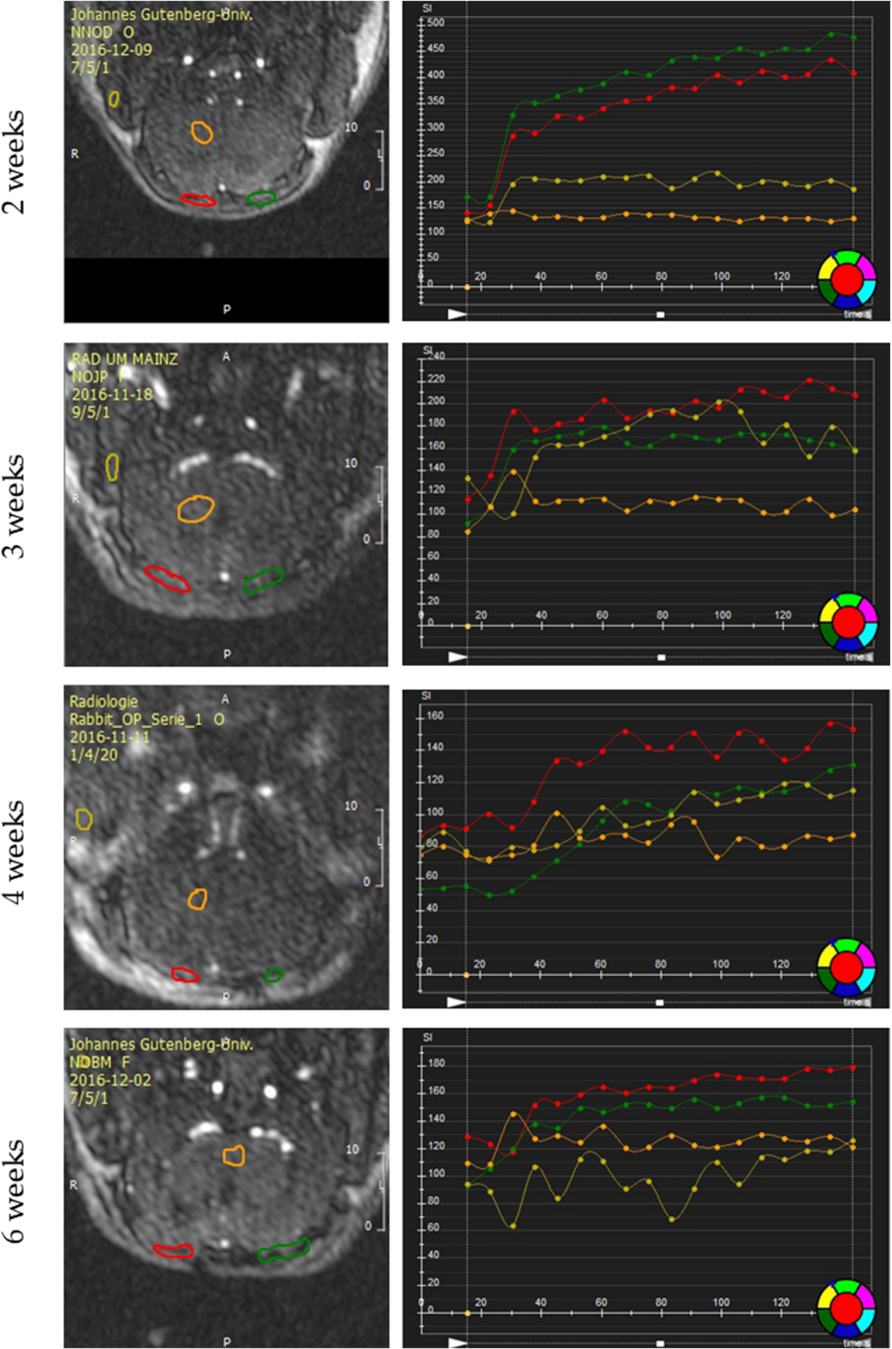
A coronal slice from one animal from each group was selected for visual comparison among different time points. The 5th slice from anterior to posterior was picked for each animal, corresponding to the center of the defect. On the right, the perfusion curves can be seen. In red, values for the right defect; in green, for the left one; in orange, for an ROI of the brain; and, in yellow, for the right masseter muscle

The defects were identifiable even prior to contrast agent injection, appearing in coronal slices as concave disk-shaped structures, being hypointense in T1w images and hyperintense in T2w ones. After contrast agent injection, the blood clot group showed a slightly more pronounced signal intensity, especially from week 3. Also, a hyperintense signal moving in a centripetal fashion was observed in both groups. In the first weeks, uptake of contrast medium was fast and washout, slow, and its distribution was concentrated in the periphery of the defect. With time, the development of a vascular network led to a more sustainable uptake and washout and to a more even distribution of the contrast agent inside the defect. A comparison between animals from each group is presented in Fig. 4.

#### IAUC_140_

**Fig. 5:**
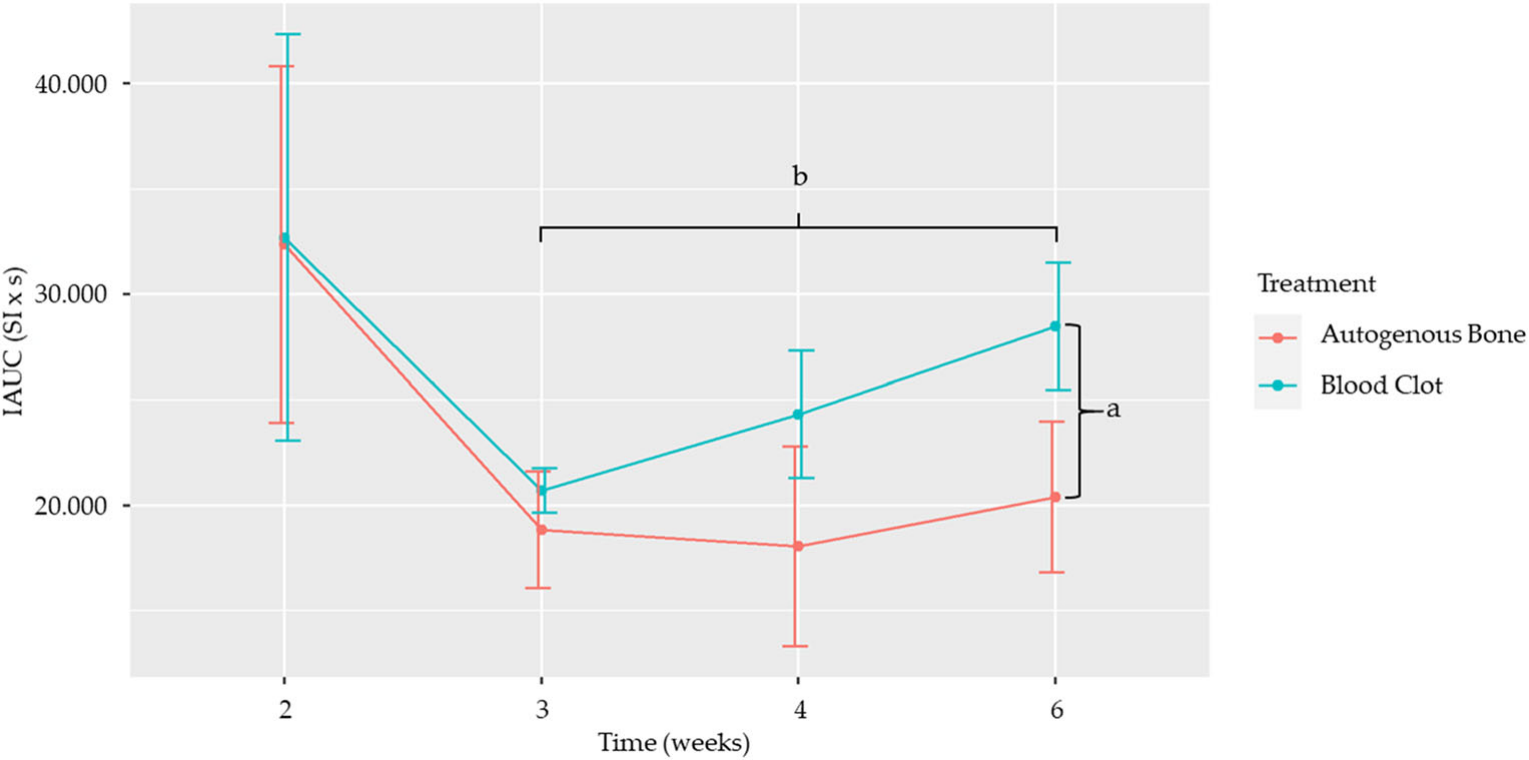
Effect of treatment on IAUC_140_ over time. Dots represent the mean value, while whiskers represent the standard error. Letters represent statistically significant differences, as seen in Tables 2 and 3

Statistically significant differences were found, when comparing treatments, at 6 weeks, when blood clot showed a statistically higher mean IAUC_140_ than autogenous bone (*p* = 0.017) (Table 2 and Fig. 5), and, when comparing time points, in the blood clot group, between weeks 3 and 6 (*p* = 0.029) (Table 3 and Fig. 5).

**Table 2:** Comparison of IAUC_140_ between the two treatments at different time points

Time (weeks)	Estimate	Standard error	*p* value
2	−331	1393	0.811
3	−1862	2572	0.469
4	−6257	2921	0.032
6	−8107	3422	0.017^a^

Statistically significant differences in IAUC140 between the two treatments at different time points are represented by letters. a =0.017

**Table 3:** Comparison of IAUC_140_ between time points, according to treatment

Time (weeks)	Treatment	Estimate	Standard error	*p* value
2–3	Autogenous bone	13,540	7310	0.383
2–4	Autogenous bone	14,136	8034	0.448
2–6	Autogenous bone	11,978	7577	0.683
3–4	Autogenous bone	776	4749	1.000
3–6	Autogenous bone	−1562	3926	1.000
4–6	Autogenous bone	−2337	5150	1.000
2–3	Blood clot	12,009	7929	0.779
2–4	Blood clot	8390	8301	1.000
2–6	Blood clot	4202	8301	1.000
3–4	Blood clot	−3619	2775	1.000
3–6	Blood clot	−7087	2774	0.029^b^
4–6	Blood clot	−4188	3705	1.000

Statistically significant differences in IAUC140 between time points, according to treatment, are represented by letters. b = 0.029

### Micro-computed tomography

#### Bone volume fraction

**Fig. 6:**
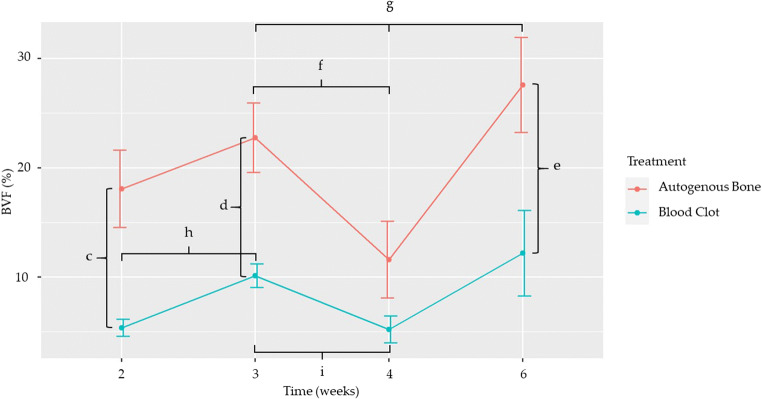
Effect of treatment on bone volume fraction over time. Dots represent the mean value, while whiskers represent the standard error. Letters represent statistically significant differences, as seen in Tables 4 and 5

Statistically significant differences were found, when comparing treatments, at weeks 3, 4, and 6, when autogenous bone showed a statistically higher mean BVF than blood clot (*p* < 0.001, *p* < 0.001, *p* = 0.003, respectively) (Table 4 and Fig. 6), and, when comparing time points, in the autogenous bone group, between weeks 3 and 4 (*p* = 0.039) and 4 and 6 (*p* = 0.005). As for the blood clot group, statistically significant differences were found between weeks 2 and 3 (*p* < 0.001) and 3 and 4 (*p* = 0.003) (Table 5 and Fig. 6).

**Table 4:** Comparison of BVF between the two treatments at different time points

Time (weeks)	Estimate	Standard error	*p* value
2	0.127	0.032	< 0.001^c^
3	0.126	0.027	< 0.001^d^
4	0.063	0.035	0.069
6	0.154	0.052	0.003^e^

Statistically significant differences in BVF between the two treatments at different time points are represented by letters. c, d < 0.001

**Table 5:** Comparison of BVF between time points, according to treatment

Time (weeks)	Treatment	Estimate	Standard error	*p* value
2–3	Autogenous bone	−0.046	0.041	1.000
2–4	Autogenous bone	0.064	0.043	0.806
2–6	Autogenous bone	−0.095	0.048	0.298
3–4	Autogenous bone	0.111	0.041	0.039^f^
3–6	Autogenous bone	−0.048	0.046	1.000
4–6	Autogenous bone	−0.159	0.048	0.005^g^
2–3	Blood clot	−0.047	0.011	< 0.001^h^
2–4	Blood clot	0.001	0.012	1.000
2–6	Blood clot	−0.068	0.034	0.288
3–4	Blood clot	0.049	0.014	0.003^i^
3–6	Blood clot	−0.020	0.035	1.000
4–6	Blood clot	−0.069	0.035	0.298

Statistically significant differences in BVF between time points, according to treatment, are represented by letters. g = 0.005, h < 0.001, i = 0.003

### Histological analysis

#### New bone formation

**Fig. 7:**
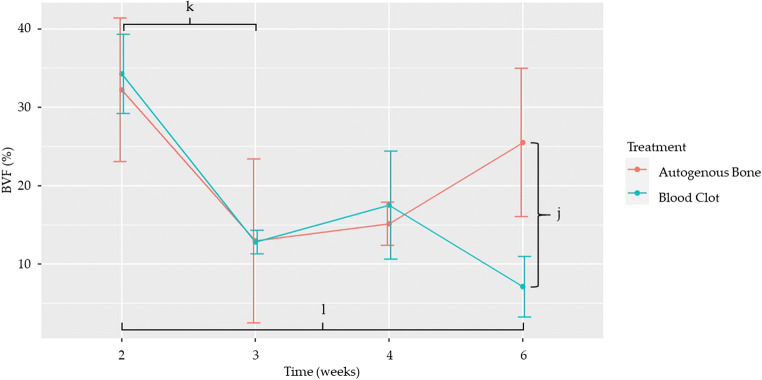
Effect of treatment on new bone formation over time. Dots represent the mean value, while whiskers represent the standard error. Letters represent statistically significant differences, as seen in Tables 6 and 7

Statistically significant differences were found, when comparing treatments, at 6 weeks, when autogenous bone showed a statistically higher mean NBF than blood clot (*p* = 0.023) (Table 6 and Fig. 7); and, when comparing time points, in the blood clot group, between weeks 2 and 3 (*p* = < 0.001) and 2 and 6 (*p* = < 0.001) (Table 7 and Fig. 7).

**Table 6:** Comparison of NBF between the two treatments at different time points

Time (weeks)	Estimate	Standard error	*p* value
2	−2.03	3.36	0.545
3	0.157	8.47	0.985
4	−2.38	7.04	0.735
6	18.4	8.12	0.023^j^

Statistically significant differences in NBF between the two treatments at different time points are represented by letters. j = 0.023

**Table 7:** Comparison of NBF between time points, according to treatment

Time (weeks)	Treatment	Estimate	Standard error	*p* value
2–3	Autogenous bone	19.28	10.52	0.401
2–4	Autogenous bone	17.09	7.82	0.1729
2–6	Autogenous bone	6.74	10.75	1.000
3–4	Autogenous bone	−2.19	7.74	1.000
3–6	Autogenous bone	−12.54	10.69	1.000
4–6	Autogenous bone	−10.35	8.04	1.000
2–3	Blood clot	21.47	4.26	< 0.001^k^
2–4	Blood clot	16.74	6.98	0.098
2–6	Blood clot	27.14	5.19	< 0.001^l^
3–4	Blood clot	−4.73	5.73	1.000
3–6	Blood clot	5.68	3.33	0.530
4–6	Blood clot	10.40	6.45	0.640

Statistically significant differences in NBF between time points, according to treatment, are represented by letters. k, l < 0.001

### Correlations

**Fig. 8:**
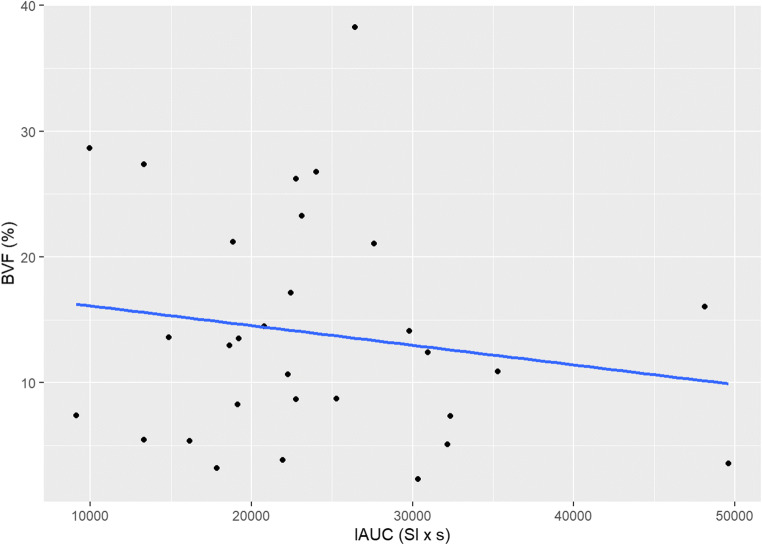
Correlation between μ-CT and DCE-MRI findings

**Fig. 9:**
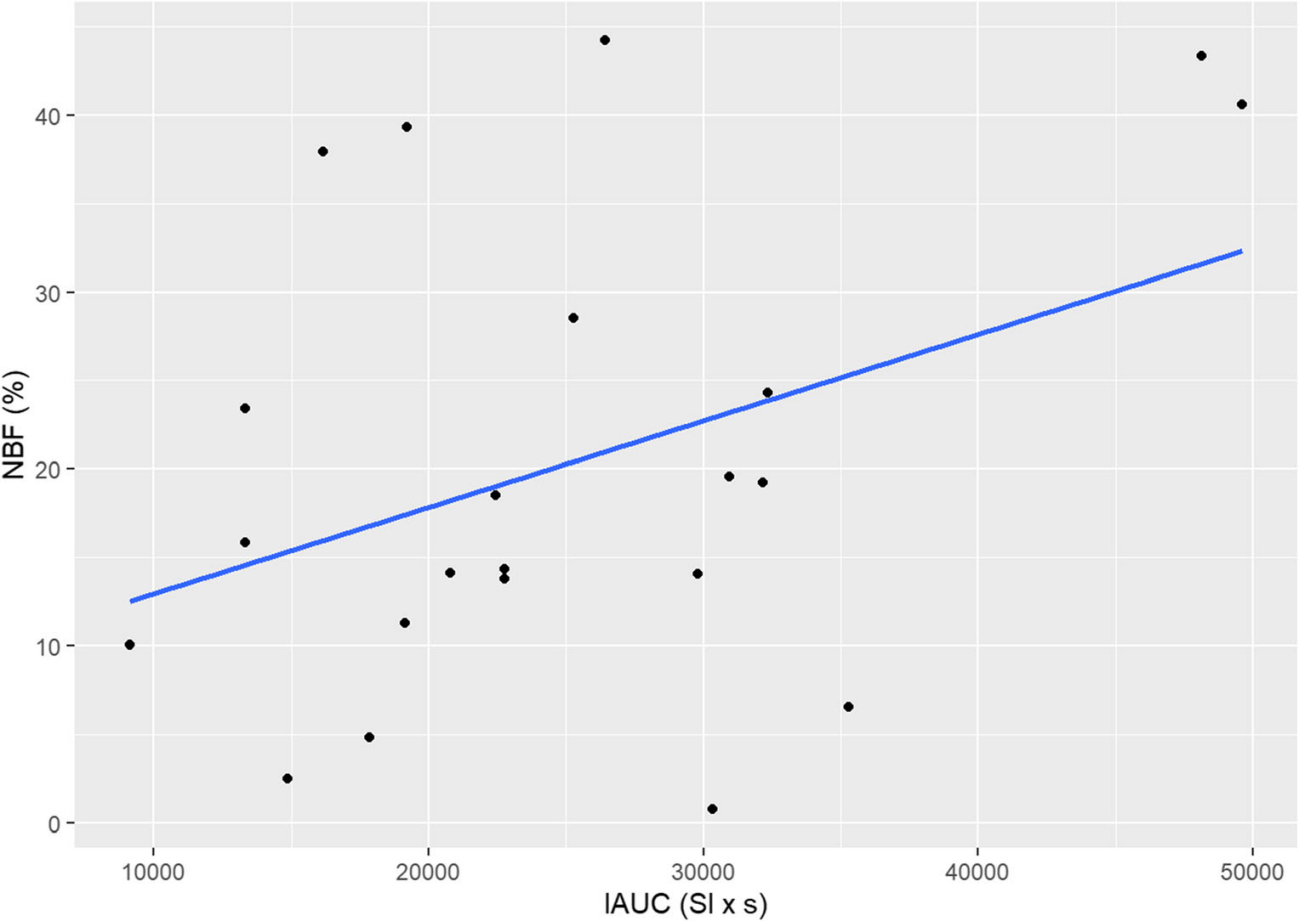
Correlation between Histology and DCE-MRI findings

**Fig. 10:**
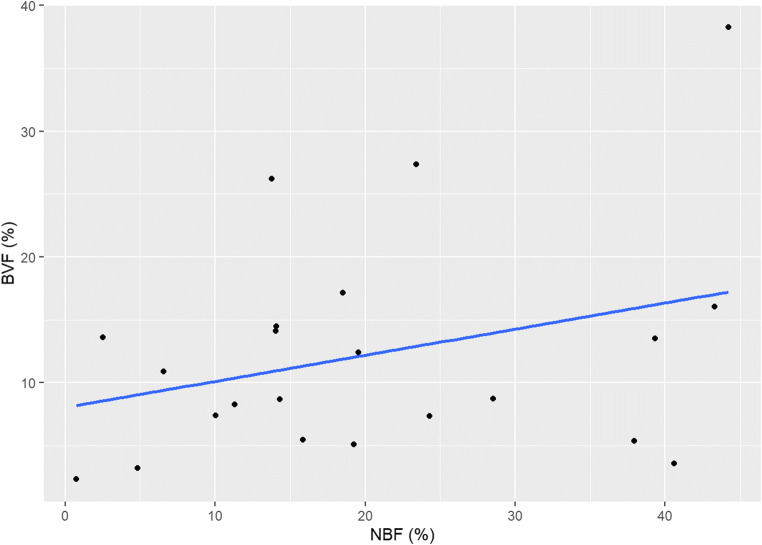
Correlation between μ-CT and Histology findings

There was no statistically significant correlation between μ-CT and DCE-MRI (*r* =−0.101, 95% CI [−0.445; 0.268]), and histology and DCE-MRI (*r* = 0.305, 95% CI [−0.133; 0.644]), nor between μ-CT and histology (*r* = 0.237, 95% CI [−0.204; 0.599]) (Figs. 8, 9, and 10).

**Fig. 11:**
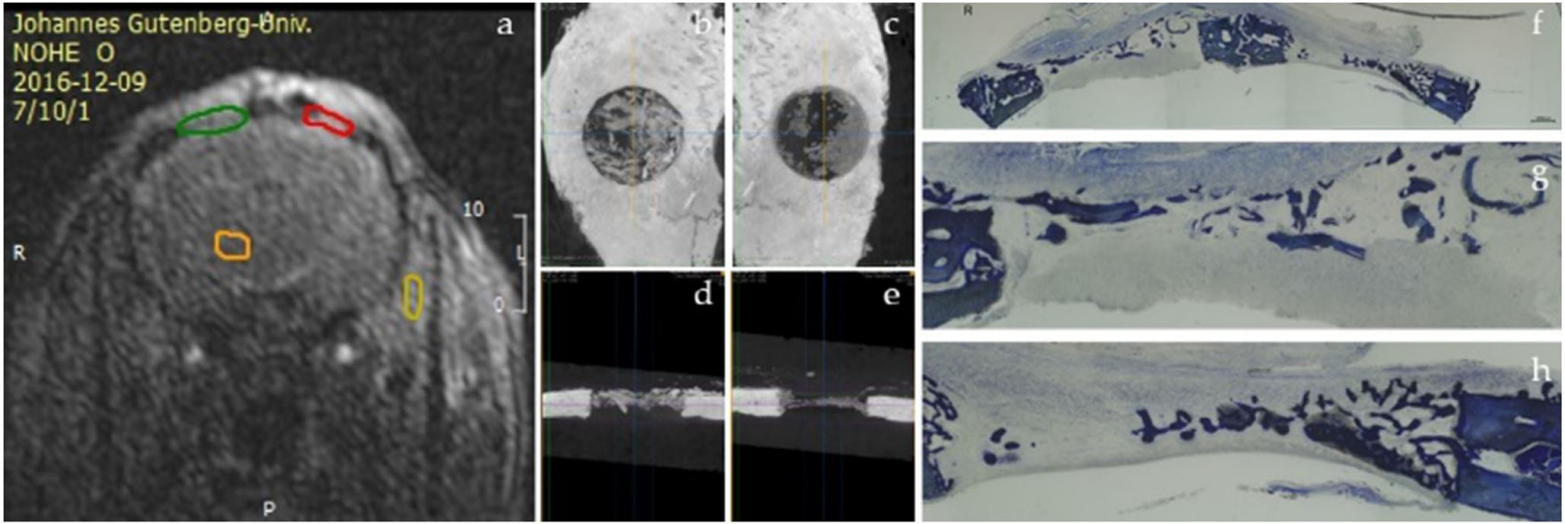
Animal from group 2 weeks selected for visual comparison among the three assessment tools. **a** Coronal slice of DCE-MRI. Highlighted in green is the right defect, which was filled with an autogenous bone; in red, the left defect, filled with blood; in orange, an ROI of the brain, and in yellow, one of the masseter muscle, which was used as references to normalize the IAUC_140_ values for both defects; **b** a bird’s view of the right and **c** left defects on μ-CT. **d** A coronal slice of the right and **e** left defects on μ-CT. **f** A ×2 magnification of a slide containing both defects, stained with toluidine blue; **g** a close-up of the right and **h** left defects

In Fig. 11, a montage is presented for visual comparison among the three assessment tools.

## Discussion

In this viability study, neovascularization could be successfully monitored during bone regeneration using DCE-MRI. We were able to consistently identify the two craniotomies and measure the blood perfusion within them.

The blood clot group showed a higher mean IAUC_140_ throughout the experiment, even though a statistically significant difference between treatments was only observed at 6 weeks. One possible explanation for this phenomenon could be the higher amount of empty space in the defects left to fill with blood, leaving more room for blood vessels to form. Also, this group lacks the osteoconductive, osteoinductive, and osteogenic capacities of autogenous bone, depending entirely on the surrounding host bone and periosteum for repair, which may trigger a longer and more pronounced inflammatory response.

Regarding the time of euthanasia, a statistically significant difference was found within the blood clot group between 3 and 6 weeks, while none was seen for autogenous bone. On a closer inspection, a trend can be observed. Between the second and third weeks, a sharp decrease in IAUC_140_ was seen in both treatment groups, consistent with a transition from the inflammatory to the proliferative phase of wound healing. After that, a steady growth was observed in the blood clot group until week 6, while in the autogenous bone group there was a slight decrease between weeks 3 and 4, after which point a growth was also observed, albeit not as pronounced as in the blood clot group. These findings contrast with Beaumont et al. [15], who observed a fast drop in perfusion from week 1 to 2 and, then, a stabilization until week 6, after which point perfusion dropped steadily until week 12, the latter being attributed to bone remodeling. Our follow-up was not as long, but a similar tendency from week 6 could be anticipated, since, as the authors point out, that is consistent with established models of bone regeneration [16]. Despite sharing the same animal model and one assessment method, the DCE-MRI, the studies differ on the grafting material and how the IAUC was calculated, which could account for these differences.

As for correlations between findings of the three assessment tools, no significant correlation was observed between neovascularization and bone regeneration, contradicting the premise that IAUC_140_ could be a biomarker of the efficacy of osteoplastic materials. Woloszyk et al. [17], however, advocate otherwise. In a study using multimodal MRI and μ-CT to quantify angiogenesis in eggs implanted with two different types of bone substitutes, the authors found that perfusion capacity (MRI) and total vessel volume (μ-CT) strongly correlated for both biomaterials, thereby their approach would be suitable to evaluate vascularization patterns and the efficacy of biomaterials. However, bone formation was not evaluated in the study, and thus, it is not possible to infer the efficacy of bone substitutes.

Coming back to our correlations, a positive correlation between DCE-MRI and histomorphometry was observed, albeit not statistically significant, which could be due to the small size of our sample. That is in line with Sauerbier et al. [18], who observed a correlation between histomorphometry findings on bone regeneration and DCE-MRI findings on neovascularization in a patient submitted to bilateral sinus lifting.

Moving on to an analysis of strong and week points, one strength of the present study is that it used the gold standard material for grafting, autogenous bone, unlike similar studies that used DCE-MRI to monitor neovascularization during bone regeneration [15, 17, 18]. These studies evaluated not only an experimental imaging method but also an experimental grafting material, which adds to the uncertainty and complicates the interpretation of findings. However, using autogenous bone turned out to be a double-edged sword, since both in the histomorphometric and μ-CT analyses, it was difficult to differentiate between grafted bones and newly formed ones. Another strong point of our study was having a negative control group, so that the natural history of bone repair could be established in DCE-MRI, creating a base for comparison for the testing of grafting materials.

Speculating on how our findings could impact clinical practice, it is reasonable to recommend DCE-MRI not as a substitute, but as a complement of CT, as a means to evaluate bone viability, especially in cases of questionable bone quality, like prior to implant placement after bone augmentation or when a pseudoarthrosis is suspected after fracture reduction.

To plan the placement of a dental implant, a panoramic radiography or a cone beam computed tomography is needed to assess bone availability. However, these examinations provide little information on bone quality. In areas where bone augmentation has been performed, it can be hard to differentiate between grafting material and newly formed bone, which can lead to an erroneous assessment of bone viability. Ideally, prior to implant placement, a histological analysis of the bone should be performed in such cases, but this is often unfeasible, since it would result in another surgical procedure. Thus, implants occasionally end up being placed in areas of questionable bone quality, putting osseointegration at risk. What our results suggest is that, in such cases, a DCE-MRI could be performed additionally to the CBCT to evaluate neovascularization where implant placement is planned, providing information on the viability of bone, allowing clinicians to make a better-informed decision, thereby making the treatment more predictable. Sauerbier et al. [18] show that this is feasible and, even though only one patient was evaluated, their results support our recommendation.

DCE-MRI could also facilitate decision-making when pseudoarthrosis is suspected and the surgeon must decide whether to reintervene or not. This is a clinical-radiological diagnosis, which can be hard to establish, especially when the information from the two examinations is contradictory, e.g., a fracture line can no longer be identified on the X-ray, but the patient still feels pain or there still seems to be mobility between fragments. Should we be able to see signs of vessels sprouting from the proximal to the distal segment, bridging the gap, which would be another indicator of bone union.

As for repercussions on pre-clinical research, our study adds to a growing body of evidence that shows that MRI is effective in monitoring neovascularization during bone regeneration in different animal models [15, 17, 19, 20]. Thus, future studies that aim at assessing the vascularization of bone grafts or bone tissue–engineered constructs can do so using MRI instead of histology. That could not only lead to a reduction in sample size and, with it, in costs, but also allow for an intra-individual longitudinal assessment.

## Conclusions

DCE-MRI consistently allowed the identification of the craniotomies and the assessment of the blood perfusion of the osteoid within them. Nonetheless, these findings did not correlate to the formation of new bone on histomorphometry or μ-CT, which speaks against the use of IAUC_140_ as a surrogate for the efficacy of osteoplastic materials. Though we showed this method to be viable, for the validation of DCE-MRI for monitoring neovascularization during bone regeneration, and, possibly, of IAUC_140_ as a surrogate for the efficacy of osteoplastic materials, confirmatory studies are needed.
